# Multimorbidity in Heart Failure: Leveraging Cluster Analysis to Guide Tailored Treatment Strategies

**DOI:** 10.1007/s11897-023-00626-w

**Published:** 2023-09-02

**Authors:** Mariëlle C. van de Veerdonk, Gianluigi Savarese, M. Louis Handoko, Joline W.J. Beulens, Folkert Asselbergs, Alicia Uijl

**Affiliations:** 1 Department of Cardiology, Amsterdam University Medical Centers, Amsterdam Cardiovascular Sciences, University of Amsterdam, Meibergdreef 9, 1105 AZ Amsterdam, The Netherlands; 2 Department of Cardiology, Amsterdam University Medical Centers, Amsterdam Cardiovascular Sciences, Vrije Universiteit Amsterdam, Meibergdreef 9, 1105 AZ Amsterdam, The Netherlands; 3 Division of Cardiology, Department of Medicine, Karolinska Institutet, Stockholm, Sweden; 4 Department of Epidemiology and Data Science, Amsterdam University Medical Centers, Vrije Universiteit Amsterdam, Amsterdam Public Health Institute, Amsterdam, The Netherlands; 5 Julius Center for Health Sciences and Primary Care, University Medical Center Utrecht, Utrecht, The Netherlands; 6 Amsterdam Public Health Research Institute, Amsterdam, The Netherlands; 7 Health Data Research UK London, Institute for Health Informatics, University College London, London, UK

**Keywords:** Heart failure, Machine learning, Clustering, Phenotyping, Precision medicine, Treatment response

## Abstract

**Review Purpose:**

This review summarises key findings on treatment effects within phenotypical clusters of patients with heart failure (HF), making a distinction between patients with preserved ejection fraction (HFpEF) and reduced ejection fraction (HFrEF).

**Findings:**

Treatment response differed among clusters; ACE inhibitors were beneficial in all HFrEF phenotypes, while only some studies show similar beneficial prognostic effects in HFpEF patients. Beta-blockers had favourable effects in all HFrEF patients but not in HFpEF phenotypes and tended to worsen prognosis in older, cardiorenal patients. Mineralocorticoid receptor antagonists had more favourable prognostic effects in young, obese males and metabolic HFpEF patients. While a phenotype-guided approach is a promising solution for individualised treatment strategies, there are several aspects that still require improvements before such an approach could be implemented in clinical practice.

**Summary:**

Stronger evidence from clinical trials and real-world data may assist in establishing a phenotype-guided treatment approach for patient with HF in the future.

## Introduction

Heart failure (HF) is a complex clinical syndrome that has been characterized as a global pandemic. According to the Global Burden of Diseases in 2017, there were an estimated 64.3 million prevalent HF patients worldwide [1]. Despite advances in evidence-based treatment of patients with HF, the disease is still paired with a substantial morbidity and mortality, with a 5-year mortality rate of 60% [2, 3]. In addition, 60% of patients are readmitted within 1 year after their initial diagnosis of HF, of which almost one-third have HF as primary cause of hospitalization [3, 4]

The goals of medical therapies for HF are to reduce symptoms, improve quality of life, prevent recurrent hospitalisations for HF, halt or reverse deterioration of cardiac function, and improve survival [5]. Patients are treated with a “one-size-fits-all” approach in which all therapies are considered for all patients following the guidelines with a selection based on ejection fraction (EF) and comorbidities.

To date, this approach has worked well in patients with HF and reduced EF (HFrEF; EF ≤ 40%). However, treatment implementation in daily clinical practice has been suboptimal [6, 7]. It is suggested that there could be a benefit from personalisation of treatment sequencing for patients with HFrEF to accomplish more effective treatment [8•], which could potentially be achieved via patient phenotyping.

The “one-size-fits-all” approach seems less fruitful in patients with HF and preserved ejection fraction (HFpEF; EF ≥ 50%), where to date, only sodium-glucose co-transporter 2 inhibitors (SGLT2i) have shown benefit [9, 10]. Perhaps, it is not the drugs that are ineffective, but rather it is the enormous heterogeneity of the patient population that predisposes the clinical trials to disappointing results in HFpEF [11, 12]. Patient phenotyping to personalise therapy has therefore frequently been suggested to disentangle the heterogeneity of the patient population.

To personalise therapies, several studies have investigated cluster analyses to discover distinct subgroups of HF patients based on their characteristics. This has led to a proliferation of clustering studies, different phenogroups based on comorbidity profiles, and different hypotheses on the origin of these clusters. Thus far, there have been no implications for daily clinical practice and how patients are treated based on clustering studies. This review therefore summarises key findings on treatment effects within phenotypical clusters of HF patients, making a distinction between patients with HFpEF and HFrEF. In addition, future directions with regard to a “phenotype-guided” treatment approach will be discussed.

## Hypothesis for a Phenotype-Guided Approach

The current foundations for HFrEF treatment consist of modulation of the renin-angiotensin-aldosterone system and the sympathetic nervous system by angiotensin-converting enzyme (ACE)-inhibitors, angiotensin receptor neprilysin inhibitors (ARNI), beta-blockers, mineralocorticoid receptor antagonists (MRA), and SGLT2i [5]. All treatments have shown to improve symptoms and survival and reduce the number of hospitalisations [5]. SGLT2i have most recently been included as they have shown to improve cardiovascular mortality, reduce HF symptoms, and improve quality of life [13, 14]. The benefit of applying comprehensive combination therapy (including ARNI/MRA/beta-blocker/SGLT2i) instead of single-agent or dual agent therapies of the most commonly used agents (i.e. ACE-inhibitors and/or beta-blockers) has been demonstrated in meta-analyses [15, 16] and was suggested in analyses from three clinical trials (PARADIGM-HF, EMPHASIS-HF, and DAPA-HF) [13, 17, 18].

Despite the overwhelming evidence from clinical trials, real-world data suggest that the implementation in daily practice is falling behind. Patients do not meet target doses, there is clinical inertia, or there are concerns with tolerability in those with impaired renal function, anaemia, atrial fibrillation (AF), lung and liver disease, or hyperkalaemia [6, 7]. Although the prevalence of comorbidities in HF clinical trials has increased over time, inclusion of patients with these comorbidities remain limited, complicating the application of evidence to individual patients [19]. Adjusting a priority or sequence in the available guideline directed medical therapies (GDMT) could be an option to take into account patient comorbidities in daily clinical practice. Currently, prioritising or sequencing of GDMT is lacking or done according to the “historical” approach, starting with an ACE-inhibitor or ARNI first, beta-blocker second, MRA third, and SGLT2i last [20•]. However, this might not be the most effective sequence for all patients, especially those that are older or have multiple comorbidities. It might not be possible to start all therapies simultaneous and therefore tailoring GDMT with a priority or sequence is needed, potentially guided by patient phenotyping.

On the other hand, in HFpEF, SGLT2i is the only therapy that has demonstrated benefit [9, 10]. Most clinical trials in HFpEF have shown neutral results, with subsequent post hoc analyses identifying potential treatment effects in specific subgroups of patients [21, 22]. Heterogeneity has been proposed as the cause for the inconclusive trial results as it could have led to a dilution of potential beneficial treatment effects.

A more nuanced classification beyond EF is likely to have positive implications for individualised patient care and clinical trial design. In 2018, Ahmad et al. already showed an improved prognostication beyond EF with distinct clinical subgroup and heterogeneity in the treatment response in a large cohort of HF patients in the Swedish HF registry using cluster analysis [23].

To elucidate a phenotype-guided approach for patients with HF, phenotyping studies use unsupervised clustering for the classifications of patients according to discriminating factors, including demographics, comorbidities, laboratory parameters, or echocardiography features. As unknown or complex relationships between these variables do not need to be pre-specified, unsupervised clustering is especially suitable to discover subgroups in a heterogeneous patient population.

Phenotyping models are data driven; therefore, results are highly dependent on the input. This was also seen in a recent systematic review, which found 34 studies that clustered patients with HF, including 19 studies in patients with HFpEF [24•]. Several clusters were observed in a multitude of studies, and, in total, nine phenogroups could be discerned. This review will focus on treatment response in the most commonly identified phenotypes across all clustering studies in HF: (1) young-low comorbidity burden; (2) metabolic; (3) AF; (4) cardiorenal; and (5) ischaemic. These phenotypes occurred in at least half of the studies described in the systematic review; the young-low comorbidity cluster was described most often (90%) and found equally in both HFpEF and HFrEF patients. Most common clusters in patients with HFpEF were the metabolic (89%) and cardiorenal (53%) clusters, while the AF (57%) and ischaemic (57%) cluster were more prevalent in patients with HFrEF [24•].

### Young-Low Comorbidity Burden Phenotype

The young-low comorbidity phenogroup is often characterised by a younger age compared to other clusters and the lack of significant comorbidities or cardiac remodelling. Cluster studies have shown that these patients have better prognosis trends and in multiple studies obesity was highly prevalent in this phenotype [25–27].

In patients with HFpEF, no therapeutic effects of ACE inhibitors/angiotensin receptor blocker (ARB)/beta-blockers were found in young patients with a low comorbidity burden [28–30]. On the other hand, two clustering substudies of TOPCAT investigating spironolactone found that MRA had favourable effects on the primary outcome of composite cardiovascular mortality and HF hospitalisation in the young-low comorbidity burden phenotype, which included subgroups of patients with a lower burden of cardiovascular remodelling, lower neurohormonal stress (i.e. low levels of N-terminal pro-B-type natriuretic peptide [NT-proBNP]), high BM,I and low burden of comorbidities [31, 32]. These findings are in line with an earlier TOPCAT post hoc study that showed that spironolactone benefits were greater in patients with low NT-proBNP [33]. These results suggest that a more modifiable substrate state is amenable to favourable changes with spironolactone. Another potential explanation for this finding might be related to the preserved renal function in this cluster, as it has previously been shown that renal dysfunction is associated with lower MRA dosage and more frequent treatment discontinuation [34, 35]. On the other hand, another substudy of TOPCAT did not observe favourable therapeutic effects of MRA in a cluster with young, smoking HFpEF-patients without significant cardiac remodelling [36].

Lastly, one study showed that combination therapy with any two treatments (ACE-inhibitors/ARB/beta-blockers/MRA/hydralazine nitrate) in patients with HFpEF was associated with lower HF hospitalisation rates in a cluster with lower comorbidity burden [37].

In patients with HFrEF, less evidence for a favourable treatment response in the young-low comorbidity cluster is seen. Some studies found favourable effects of ACE-inhibitors in young, obese patients, but this group had overlapping characteristics with the ischemic phenotype [23, 38]. In contrast, Tromp et al. did not observe favourable effects in young HFrEF patients [26].

Conflicting results have been found with respect to beta-blockers. One study including all patients with HF showed that there was a drug interaction with beta-blockers and the young-low comorbidity cluster with a more favourable response [23]. This cluster included more patients with lower EF, were more often male, and had higher BMI. Comparable to the studies in TOPCAT, this cluster also had lower NT-proBNP levels [23]. Other studies did not find beneficial effects of beta-blockers in this cluster [25, 26, 38].

In addition, in HFrEF patients, conflicting therapeutic results of MRA-treatment were found in this young, obese phenotype [23, 38].

Currently, no SGLT2i or ARNI clustering studies have been performed across the EF spectrum, so it remains to be elucidated whether there are differences in treatment response between patient clusters for these therapies.

### Metabolic Phenotype

In patients with the metabolic phenotype, comorbidities such as diabetes mellitus (DM), hypertension and hypercholesterolemia play an important role by inducing systemic inflammation that may lead to cardiac remodelling and fibrosis [39, 40]. Therefore, therapies aiming to reduce this pro-inflammatory state may be of great importance and comprises of three pillars: (1) secondary prevention by lifestyle based interventions such as exercise training and changes in nutrition [41]; (2) management of risk-factors and comorbidities [5]; and (3) HF medical therapies that target the pro-inflammatory state and improve cardiac remodelling.

In HFpEF, both Kao et al. and Gu et al. showed that in a metabolic cluster, ACE inhibitors and/or ARB were associated with a lower risk of all-cause mortality [28, 29]. However, many of these metabolic phenotype patients showed overlapping characteristics with other phenotypes, including the cardiorenal and ischemic phenotype, respectively [28, 29].

Both in HFpEF and HFrEF, beneficial effects of MRA in the metabolic cluster were demonstrated [36, 38]. This might be explained by the favourable effects of MRA on cardiac remodelling since aldosterone inhibition might lead to reductions of collagen and extracellular matrix, less fibrosis, and reduced myocardial stiffness resulting in improved LV diastolic and systolic function [42].

Furthermore, conflicting results with respect to beta-blockers have been shown in HFpEF patients, whereas beneficial effects in HFrEF patients with metabolic comorbidities were found [29, 37, 38].

One study clustered DM type 2 patients from the EMPA-REG OUTCOME tria;, this study showed that SGLT2i were beneficial for all clusters consisting of patients with DM type 2 and (1) younger age with preserved kidney function, (2) females with limited coronary artery disease, or (3) older patients with severe coronary artery disease and renal insufficiency. There was no interaction between cluster membership and SGLT2i use [43]. It is likely that SGLT2i will be beneficial for all HF phenotypes and be of specific importance in patients with cardiometabolic abnormalities, since it does not only improve HF but also leads to better glucose regulation in DM type 2 patients. In addition, in a well-phenotyped HFpEF population, it was found that SGLT2i-treatment resulted in clinical relevant weight reduction, lowering of systolic blood pressure, and improved exercise capacity. Similar treatment effects were observed in HFpEF patients with and without DM type 2 [44].

### AF Phenotype

AF is often included as a component in clustering studies and can cluster within a variation of AF phenotypes, i.e. elderly AF, female AF, and hypertensive AF, which could be related to the difference in pathophysiology between HFrEF and HFpEF. AF is more often seen as a consequence of HF in HFrEF, while in HFpEF, it is proposed that both ventricular and atrial myopathy may develop in parallel [45, 46].

In HFpEF, Sotomi et al. found no significant efficacy of HF-medications that target anti-inflammatory or neurohormonal remodelling in this patient group. Instead, aggressive rhythm control (catheter ablation and/or antiarrhythmic drugs) may benefit AF patients [30]. Other studies also did not find any beneficial differences in treatment response for ACE inhibitors, ARB, beta-blockers, or MRA in this cluster [28, 29, 36].

In HFrEF, a large clustering study based on 11 beta-blocker randomised controlled trials (RCTs) showed that beta-blockers did not reduce all-cause mortality in the overall AF group, but rather the authors found a beneficial treatment response in younger, HFrEF patients in an AF cluster [27•]. Survival was better in this specific AF cluster and might be explained by a less severe phenotype and fewer comorbidities compared to the other AF cluster subgroups [27•].

In general, AF may occur as a primary or secondary phenomenon in HF. Therefore, AF patients may also be represented in the other HF phenotypes that may respond better to HF medications.

### Cardiorenal Phenotype

AF was also a recurring factor in many studies that identify a cardiorenal phenotype. This phenotype represents a group of frail patients, with a high prevalence of worse kidney function and chronic kidney disease (CKD), often associated with female sex. Impaired renal function often concerns cardiologists with reduced tolerance and safety in relation to treatment with HF medication [47].

Although many pivotal HF RCTs excluded patients with severe CKD, it was shown that the group of patients with the highest prevalence of CKD had the most benefit from ACE inhibitor/ARB treatment across the EF spectrum [23, 26, 28, 30]. This might be explained by the cardiorenal protective effects of these treatments [48]. However, both MRA and beta-blockers tended to have less effect in older, frail patients [26, 30, 31, 36]. Sotomi et al. even found harmful effects of beta-blockers in older frail HFpEF patients with poor nutritional status. However, these results might be underpowered; thus, careful interpretation is required [30]. Similarly, Tromp et al. found that a cluster of older HFrEF patients with anaemia and CKD did not derive treatment benefit from beta-blockers and actually might have potential harm from up-titration of beta-blockers [26].

There is a growing body of evidence that supports the efficacy and safety of SGLT2i in patients with CKD, based on complex mechanisms of action that extend far beyond glycosuria and that confer beneficial effects on cardiovascular and renal haemodynamics, fibrosis, inflammation, and end-organ protection [49]. Although there are no clustering studies available in HF patients, SGLT2i provide a major benefit in patients with CKD [50]. Although an initial kidney function dip is expected, this initial change is reversible and not associated with adverse outcomes [51, 52].

### Ischaemic Phenotype

Lastly, the ischaemic phenotype is characterised by a history of myocardial infarction (MI) resulting in damage of the cardiac contractile elements contributing to lower LVEF [53, 54]. This phenotype is more often reported in studies with HFrEF patients [23, 26, 55].

In the studies of Ahmad et al., two ischemic clusters were identified: one cluster of HFrEF patients with older age and more comorbidities and the second cluster comprised of younger HFrEF patients with few comorbidities [23, 55]. In both groups, favourable effects of ACE-inhibitors/beta-blockers were demonstrated. However, no beneficial effects of MRA were found, and the younger group did not gain benefit from ARB. Similarly, in an older subgroup with low BMI, worse kidney function, multiple comorbidities, and the highest rates of previous revascularisations (70%), no effects of spironolactone were found [38].

Similarly, biomarker analyses revealed that although the ischemic subgroup in patients with HFrEF had the highest rate of up-titration of ACE-I/ARB to recommended dose, this group experienced only neutral effects on clinical outcomes. In addition, a trend towards a beneficial effect of beta-blockers was found in the ischemic phenotype [26]. Of interest, beneficial effects of beta-blockers were not only found in HFrEF patients but also in a cluster of metabolic patients with ischemic heart disease in HFpEF patients [29]. This cluster also had benefit with respect to ACE-inhibitors which conflicts with what was seen in patients with HFrEF [29].

There are no clustering studies available that study the effects of ARNI in ischemic HF patients. PARADISE-MI showed that ARNI did not reduce the risk of new MIs of coronary vascularisation in patients with recent MIs and related LV systolic dysfunction. However, the pre-specified combined endpoint of coronary artery disease related death, hospitalisations of revascularisation was positive, with a 14% risk reduction during long-term follow-up [56]. A recent meta-analysis on the real-world evidence of ARNI for patients with HFrEF showed that real-world patients with ischaemic aetiology were receiving ARNI less often as compared to patients in the PARADIGM-HF trial, even though PARADIGM-HF showed effectiveness of ARNI irrespective of aetiology [57, 58].

## Treatment Response Heterogeneity Across Heart Failure Clusters

**Fig. 1:**
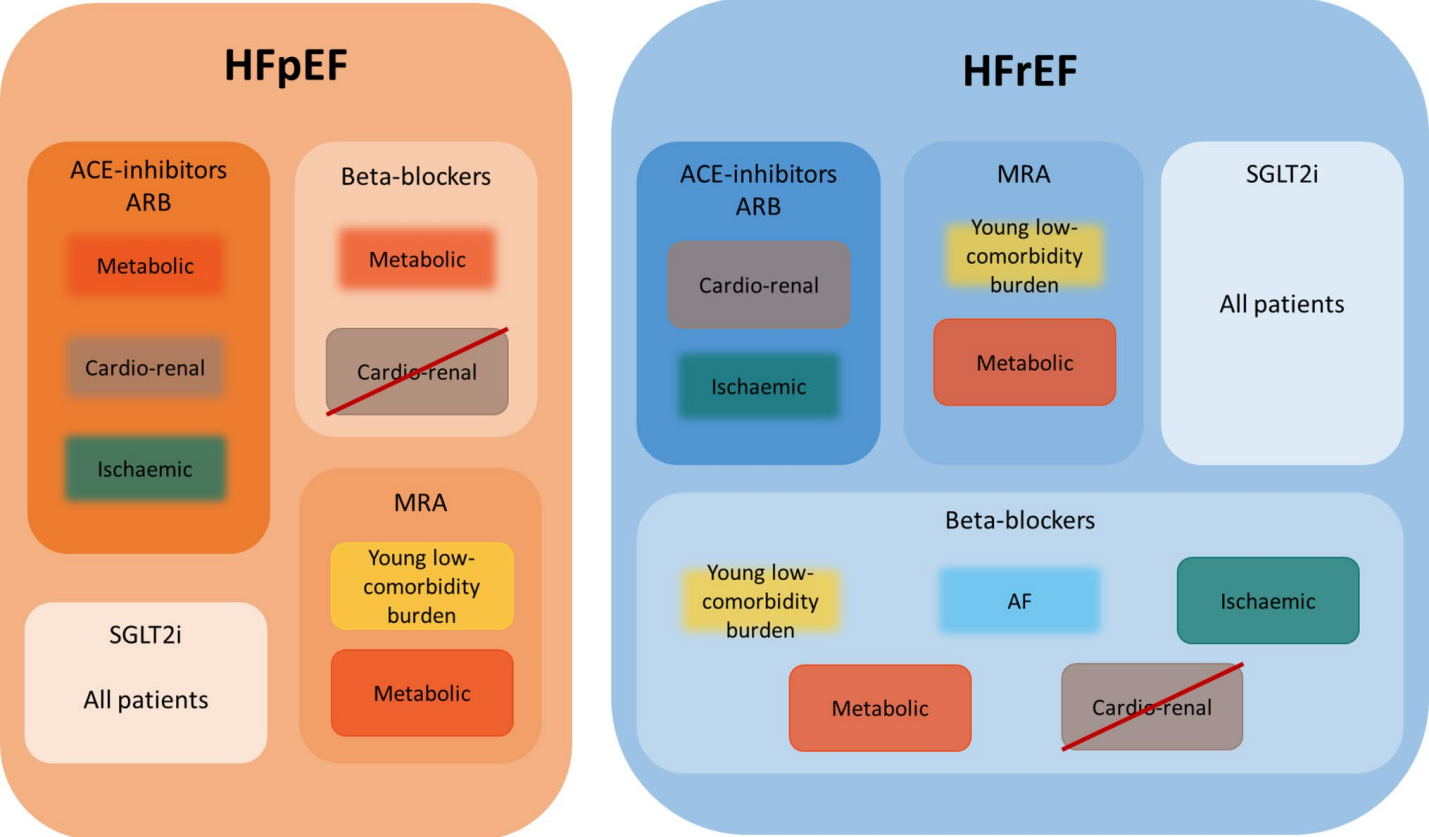
Exploring evidence of potential treatment response across phenotypical clusters in heart failure. HFpEF, heart failure with preserved ejection fraction; HFrEF, heart failure with reduced ejection fraction; ACE inhibitor, angiotensin-converting enzyme inhibitors; ARB, angiotensin receptor blocker; MRA, mineralocorticoid receptor antagonists; SGLT2i, sodium-glucose co-transporter 2 inhibitors; AF, atrial fibrillation. Blurred box borders, less or conflicting evidence for phenotype treatment response; crossed-out box, potential harm in phenotype treatment response

Cluster studies show that RAS inhibitors, such as ACE inhibitors or ARB, have beneficial effects in all HFrEF patients regardless of phenotypes types and did in general not have any favourable effects in HFpEF patients. However, ACE inhibitors may have beneficial effects in HFpEF patients with a cardiorenal phenotype and HFpEF patients with an overlapping metabolic/ischemic phenotype; however, there was conflicting evidence (Table 1, Fig. 1).

**Table 1:** Summary of findings

	HFrEF	HFpEF
All patients	All treatments beneficial for all patients, including SGLT2i and ARNi	SGLT2i beneficial for all patients
**Hypothesis for treatment effects across phenotypes**		
Young-low comorbidity	ACEi/ARB: some favourable effects in young-obese patients with overlapping characteristics from the ischaemic group for all-cause mortality [23] and a composite of cardiovascular (CV) death and heart failure (HF) hospitalisation [38]. Another study found no favourable effects on a composite outcome of all-cause mortality and HF hospitalisation [26]	ACEi/ARB: no therapeutic effects found in any of the studies [28–30]
	Beta-blockers: conflicting results with one study finding favourable effects on all-cause mortality [23] compared to others that did not on a composite outcome of all-cause mortality and HF hospitalisation [26] or all-cause mortality [27, 59]	Beta-blockers: -
	MRA: conflicting results with worse all-cause mortality in one study [23] versus favourable outcomes for the composite of CV death and HF hospitalisation in another study [38]	MRA: favourable effects on composite CV mortality and HF hospitalisation [31, 32] but not confirmed in another study [36]
Metabolic	ACEi/ARB: -	ACEi/ARB: lower risk of all-cause mortality, however there was overlap with characteristics from the cardiorenal and ischaemic phenotype, respectively [28, 29]
	Beta-blockers: favourable effects on the composite of CV death and HF hospitalisation [38]	Beta-blockers: conflicting results with no beneficial effects on HF hospitalisation in one study [37] but favourable effects found in another study for all-cause mortality and the composite of all-cause death or HF hospitalisation [29]
	MRA: favourable effects seen on the composite of CV death and HF hospitalisation [38]	MRA: favourable effects seen on the composite CV mortality and HF hospitalisation [36]
Cardiorenal	ACEi/ARB: subgroups with the highest CKD prevalence had the most benefit for all-cause mortality [23] and the composite of all-cause mortality and HF hospitalisation [26]	ACEi/ARB: subgroups with the highest CKD prevalence had the most benefit on all-cause mortality [28] and the composite of all-cause death and HF hospitalisation [30]
	Beta-blockers: one study found that in an older HFrEF cluster with anaemia and CK,D there might be potential harm from up-titration on composite of all-cause mortality and HF hospitalisation [26]	Beta-blockers: potential harmful effects in older frail patients with the composite outcome of all-cause death and HF hospitalisation [30]
	MRA: might be less effective on the composite outcome of all-cause mortality and HF hospitalisation [26]	MRA: might be less effective as was seen in studies with the composite outcome of all-cause death and HF hospitalisation [30] or CV mortality and HF hospitalisation [31, 36]
Atrial fibrillation	ACEi/ARB: -	ACEi/ARB: no favourable effects seen in any of the studies [28, 29]
	Beta-blockers: specific atrial fibrillation subgroup of young men experiencing beneficial effects on all-cause mortality [27•]	Beta-blockers: -
	MRA: -	MRA: no favourable effects on the composite outcome of CV mortality and HF hospitalisation [36]
ischaemic	ACEi/ARB: favourable effects in both older and younger cluster with ischaemic heart disease for ACEi but not ARB [23, 55] but not confirmed in another study with composite outcome of all-cause mortality and HF hospitalisation [26]	ACEi/ARB: beneficial effects seen in a metabolic cluster with ischaemic heart disease on the composite of all-cause death or HF hospitalisation [29]
	Beta-blockers: favourable effects in both older and younger cluster with ischaemic heart disease [23, 55] also confirmed in another study with the composite outcome of all-cause mortality and HF hospitalisation [26]	Beta-blockers: beneficial effects seen in a metabolic cluster with ischaemic heart disease for all-cause mortality and the composite of all-cause death or HF hospitalisation [29]
	MRA: no beneficial effects seen in any of the studies [23, 38, 55]	MRA: -

Beta-blockers had beneficial effects in HFrEF patients in sinus rhythm and are in general not effective in HFpEF. Although beta-blockers did not have favourable effects in the whole HFrEF-AF population, there was one AF subgroup consisting of young males that experienced favourable effects of beta-blockers. Results of benefit in this AF cluster should be further confirmed. In addition, beta-blockers could even be harmful in older patients with a cardiorenal phenotype, both in HFrEF and HFpEF. This potential harmful relationship should be further investigated (Table 1, Fig. 1).

Regardless of phenotypes, MRAs have shown to be effective in patients with HFrEF and showed some potential treatment effects in post hoc studies in HFpEF [22]. MRA may have specific benefit in all young, obese patients with a low-comorbidity burden and in metabolic patients with multiple comorbidities, regardless of EF. However, MRA may be less effective in the ischemic phenogroup (Table 1, Fig. 1).

Based on the positive trials in both patients with HFrEF and HFpEF, it is expected that SGLT2-i are effective in all phenogroups and may have the most beneficial effects in the metabolic and cardiorenal clusters. However, there is no evidence to date for a heterogeneous treatment response across HF phenotypes for both SGLT2-I and ARNI treatment and should be further investigated.

Although results of treatment benefit in different clustering studies are intriguing, findings from these studies should be interpreted with caution. Several studies were observational by nature and therefore prone to confounding by indication and selection bias. On the other hand, clinical trials are often underpowered to detect differences in treatment effects across subgroup analyses, let alone subgroup effects across clusters. In addition, only a handful of studies assessed statistical interaction between treatment and clusters with interaction modelling; most studies chose to stratify across clusters which does not provide a test of statistical significance on the difference between the clusters.

Moving forward, it is imperative that clinical trials a priori plan to analyse differences in treatment response across clusters so that interaction modelling can be considered for sample size calculations for a powered analysis. By taking patient clusters into account in trial planning, either by developing a cluster model or incorporating an existing model, an adequate number of participants could be recruited within each cluster to evaluate treatment response across phenotypes. In addition, phenotypes should be further validated in real-world data with real-world evidence as the diversity and heterogeneity of the HF patient population are significantly present here, with a good representation of elderly patients or those with comorbidities such as AF or CKD.

## Implementation in Clinical Practice

Cluster models have shown many similarities between studies and the discovered phenotypes, even while using different study populations, phenotyping variables, and different techniques. However, one of the limitations is that phenotypes are generally not mutually exclusive, for example, patients with AF could fall within a number of phenotypes depending on the probability of other characteristics. This hampers the consolidation of the available evidence. Therefore, current evidence is not sufficient to readily implement a phenotype-guided approach in clinical practice; however, it could be used as hypothesis generating evidence. More validation studies are necessary to understand the differences between HF phenotypes, the underlying aetiologies, and which therapies could be delivered in a more effective and targeted approach for better outcomes in patients with HF. A solution for the reproducibility and generalisability of cluster models is to readjust or fine-tune the current models with site specific information to stimulate implementation in local routine clinical care.

Most important future implications for a phenotype-guided approach in patients with HFrEF would be based on sequencing or prioritising treatment strategies based on clusters. All treatments have been proven to be effective in all patients in RCTs; however, as the treatment for HF has evolved with many (new) therapies to offer, it is clear that GDMT has become more and more complex. In addition, patients seen in daily clinical practice are often older with multiple comorbidities with potential for contraindications, adverse effects, and polypharmacy. Therefore, it is of the utmost importance to match the best treatment with more individualised patient profiles; a phenotype-guided approach could assist in this.

For patients with HFpEF it is imperative that the hypotheses regarding phenotypes can be confirmed and a heterogeneous treatment response across clusters can be validated. Implications for clinical practice could be substantial if indeed it will be established that the heterogeneity of the patient population with HFpEF has led to neutral trials in the past.

Machine learning could play an important role in using digital healthcare data to select the right therapy to improve outcomes for individual patients. Results from this review could be used as hypothesis-generating to guide clinical trial design.

## Conclusions

A heterogeneous treatment response can be seen in phenotypical clusters across the EF spectrum. While a phenotype-guided approach is a promising solution for individualised treatment strategies, there are several aspects that still require improvements before such an approach could be implemented in clinical practice. Both cluster algorithms and hypotheses for heterogeneous treatment response should be confirmed and validated in appropriately powered studies, and clinical trials should be designed a priori to take into account these validated clusters. With stronger evidence, both from clinical trials and real-world data, this may help to establish a phenotype-guided treatment approach for patients with HF in the future.
